# Similarities and Differences Matter: Considering the Influence of Gender on HIV Prevention Programs for Young Adults in an Urban HBCU

**DOI:** 10.3390/ijerph14020133

**Published:** 2017-01-29

**Authors:** Ian Lindong, Lorece Edwards, Sabriya Dennis, Olaoluwa Fajobi

**Affiliations:** School of Community Health and Policy, Morgan State University, 1700 E Cold Spring Ln, Baltimore, MD 21251, USA; Lorece.Edwards@Morgan.edu (L.E.); Sabriya.Dennis@Morgan.edu (S.D.); olfaj1@Morgan.edu (O.F.)

**Keywords:** HIV, AIDS, HBCU, African American, youth, gender, prevention

## Abstract

Human Immunodeficiency Virus/Acquired Immune Deficiency Syndrome (HIV/AIDS) disproportionately burdens African American youth and young adults. In studies conducted in Historically Black Colleges and Universities (HBCU) settings, African American youth generally perceive themselves as having a low risk of contracting HIV and sexually transmitted diseases (STDs) despite having higher rates of unprotected sexual encounters, multiple sex partners, and particularly low rates of HIV testing and awareness of HIV status. These findings position HBCUs in a pivotal role for theory-based research and practice to modify behaviors in order to decrease HIV acquisition risk. Get Students Mobilized and Retooled to Transform (SMART) is an interventional research project in an urban HBCU in a northeastern metropolitan area in the US. The project is designed to assess and then address irresponsible behavior among students on college campuses that leads to illicit drug use, excessive alcohol consumption and underage drinking, and risky sexual behaviors that increase the likelihood of acquiring HIV and STDs. As gender plays a critical role in interventions, this article explores gender similarities and differences to inform the planning and implementation of Get SMART and any subsequent projects that address substance and alcohol use and HIV in an HBCU setting. Survey research was conducted to find similar and different factors that may be valuable in implementing and tailoring evidence-based interventions in a predominantly African American campus setting. Survey results revealed that more young adult women consume alcohol and use marijuana than young adult men. Young adult men were also more likely to be tested for HIV when compared to young adult women.

## 1. Introduction

### 1.1. Background

Human Immunodeficiency Virus/Acquired Immune Deficiency Syndrome (HIV/AIDS) disproportionately burdens African American youth and young adults [1,2,3]. While African Americans are estimated to account for almost half (44%) of persons living with HIV in the US as well as of newly diagnosed cases of HIV, African American youth aged 13 to 24 represents 34% of these newly diagnosed cases, about 7000 new HIV infections added to persons living with HIV each year [3]. Young African American men who have sex with men (MSM) are at the highest risk (as much as 11 times more than young white males) and African American women are more vulnerable compared to women of any other race in that age group (as much as 20 times more than young white females) [4,5,6]. The high prevalence of HIV in the African American community alone increases the risk of contracting HIV, as African American youth are more likely to engage in sexual relations with other African Americans, sometimes without protection [7,8,9]. HIV risk in this subgroup is multifactorial; higher rates of sexually transmitted diseases (STDs), stigma, socioeconomic status, and lack of awareness of HIV status are more likely to escalate HIV acquisition rates. In fact, over 70% of HIV-infected African American MSM are unaware of their HIV status, a major factor contributing to the spread of HIV. The problem is further magnified by the low rates of care and treatment (21%) and the controlled viral load (18%) of those youth diagnosed with HIV [3]. 

### 1.2. HIV and the Historically Black Colleges and Universities Setting

Historically Black Colleges and Universities (HBCUs) have an African American student population of about 80%. Overall, HBCUs enroll 25% of African Americans seeking postsecondary education [10]. In the past decade, there has been increased attention on HBCUs because of the increased rates of HIV among students attending these institutions. In studies conducted in HBCU settings, African American youth generally perceive themselves as having a low risk of contracting HIV and STDs despite having higher rates of unprotected sexual encounters, multiple sex partners, and particularly low rates of HIV testing and awareness of HIV status [4,11,12,13]. In a study conducted at multiple HBCU sites, women had higher HIV knowledge scores than men but more men reported significantly higher usage of HIV testing services and awareness of such services in the communities. However, overall, there is no difference between genders in the perception of HIV risk as both reported a low risk of acquiring HIV [14]. Another study conducted in HBCUs revealed that high knowledge scores neither translate to preventive behavior nor accurate risk perception. Despite high knowledge scores, there was a low rate of condom use during the last sexual encounter of students who had multiple sexual partners [15]. Spontaneous sexual encounters, low risk perception, and partner-related perceptions were found to be strong predictors of non-condom use during the last sexual intercourse [16]. The low risk perception of HBCU students also manifested in the low rates of testing, despite the increased availability of HIV testing services on campus [17,18]. In a mixed method study conducted at an HBCU in the Southeast US, quantitative findings revealed high levels of perceived knowledge of HIV transmission, a low perception of risk in acquiring HIV, and continued practice of risky sexual behavior. Qualitative results showed three pervading themes for avoiding testing: scared to know HIV status, prefer to not know status, and lack of discussion about HIV [19]. 

Despite evidence of higher HIV rates and the elevated risk of acquiring HIV among students attending HBCUs, it is also important to note the environmental context surrounding HBCUs. These institutions are more likely to be located in communities where the rates for HIV and STDs are already higher than the general population. In a study of communities hosting HBCUs, it was found that the risk for a student, who more commonly has sexual partners who are not college students but live in the surrounding communities [20], in acquiring HIV or an STI from a potential sex partner increases because of the already high prevalence of these conditions in the host communities [21]. 

These findings position HBCUs in a pivotal role for theory-based research and practice to modify behaviors and effect change in the environment in order to decrease HIV acquisition risk. 

### 1.3. Get Students Mobilized and Retooled to Transform (SMART) 

Get SMART is an interventional research project in an urban HBCU in a northeastern metropolitan area in the US. The project is designed to assess and then address irresponsible behavior among students on college campuses that leads to illicit drug use, excessive alcohol consumption and underage drinking, and risky sexual behaviors which increase the likelihood of acquiring HIV and STDs. The main goal of the project is to create a sexually healthy student campus environment by systematically realigning campus priorities to include the sexual health and welfare of its student body. The project is aimed primarily at African American and minority young adults (ages 18–24) on campus including the immediate community surrounding the university.

**Figure 1:**
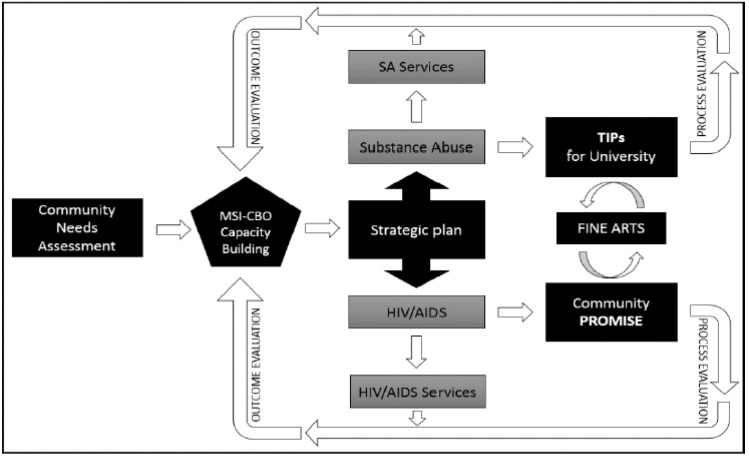
The Get Students Mobilized and Retooled to Transform (SMART) prevention framework. HIV/AIDS: Human Immunodeficiency Virus/Acquired Immune Deficiency Syndrome; MSI-CBO: Minority-Serving Institution-Community Based Organization; PROMISE: Peers Reaching Out and Modeling Intervention Strategies; SA Services: Substance Abuse Services; TIPs: Training Interventions Procedures.

The incorporation of fine arts in the intervention renders the uniqueness of the approach. Framed in a universal language through artistic expressions within two dynamic evidence-based interventions (Community Peers Reaching Out and Modeling Intervention Strategies (PROMISE) and Training Interventions Procedures (TIPs) for the university), the project tailors itself to the culture, norms, beliefs, attitudes, and practices unique to African American college students. In addition, a modified version of an American Cancer Society-designed program for smoking cessation is built into the intervention. The project takes a multidisciplinary approach to not only address the reduction of substance abuse and HIV/AIDS transmission but also the structural and social environmental determinants that drive risk-taking behaviors among young adults. Figure 1 is a depiction of the prevention framework of the project. 

### 1.4. Purpose

Gender plays a role in the effectiveness of any behavior modification program and sexual risk reduction is no different [22,23]. Although the intervention caters to young adults of both sexes, being critically conscious of the behavioral differences and similarities across gender becomes imperative in meeting gender-specific needs [24]. As the literature comparing genders on sexual behavior among college-educated African American young adults is limited, this article explores the similarities and differences to inform the planning and implementation of Get SMART and any subsequent projects that address substance and alcohol use and HIV in an HBCU setting. 

## 2. Experimental Section

### 2.1. Research Design

The initial phase of the project is a needs assessment using mixed approaches in data collection. For purposes of risk behavior assessment and this article, a survey research design was employed. The design was selected as it was deemed most appropriate and efficient in providing a valid and reliable quantification of the magnitude of risky behaviors in a university setting. Surveys, when performed correctly, may accurately depict a broad range pervasive behaviors of a given community or setting. A qualitative approach was used to supplement and further explain the survey results but its findings will not be discussed in this article. 

### 2.2. Survey Instrument

The survey instrument used is the Minority AIDS Initiative (MAI) Adult and Youth Questionnaire provided by the Substance Abuse and Mental Health Services Administration (SAMHSA). The instrument is a modular survey with four sections that cover basic demographic information and knowledge, attitudes, and practices relating to alcohol, smoking, illicit drugs, and sexual relations. The instrument represents, in its series of questions, the multifactorial nature of alcohol and substance use and sexual behavior. 

### 2.3. Participant and Procedures

Students aged 18 to 24 years were eligible to participate in the survey. The survey was advertised through the university website, student information e-mail, and health and wellness fairs. Participants were verified for eligibility using either a university-issued identification or confirmation using university student e-mail account. The MAI Adult and Youth Questionnaire was administered to participants in two modalities: (1) on-site where students complete the questionnaire in a designated classroom; and (2) online where the questionnaire is e-mailed to the participant and drops off the completed survey in a designated area. A small non-monetary incentive was handed to participants on completion of the survey for their participation in the survey. 

### 2.4. Data Analysis

A descriptive analysis was conducted on key sociodemographic factors, exposure to substances and alcohol, HIV and STD status, diagnosis, testing, and sexual behavior. A nonparametric analysis (Chi-square test) was used to test for differences between male and female young adult participants in the following variables: (1) knowledge of HIV status; (2) HIV testing; (3) use of substances and alcohol; (4) STD diagnosis; and (5) sexual behaviors. Data were analyzed using STATA v.14, (StataCorp LLC, College Station, TX, USA).

## 3. Results

There were 365 respondents to the survey study which was conducted in the first three months of the project. The respondents were predominantly black (95%) (mean age = 20 years), heterosexual (84%), and single (77%). Gay/lesbian and bisexual respondents were at 6% and 7%, respectively. A higher proportion of female respondents (56%) participated in the survey (Table 1).

Alcoholic beverage drinking (54%) was common among the respondents when asked about exposure within the last 30 days. Lifetime and three-month exposures displayed the same trend. As for substance use, marijuana (41%) was the most popular among the respondents. Thirty-one percent (31%) of the respondents claimed to have had no exposure to both alcohol and substances within the past 30 days. 

Seventy percent (70%) of the respondents were aware of their HIV status but only 32% were tested within the past three months and 29% were never tested for HIV. On occasions where HIV testing was available on-site during surveys, 77% declined to be tested. Nine percent (9%) of the respondents were diagnosed at least once with STDs. Regarding condom use during the most recent sexual encounter, 59% used condoms while 41% engaged in unprotected sex. About 40% of participants viewed sex as a coping strategy for stress related to school, work, and relationships.

Comparing across male and female genders, both were similar in terms of knowledge of HIV status, although males were more likely to be tested when the opportunity was presented (*p* < 0.001), in this case on some days while the survey was ongoing. There was no significant difference between male and female respondents on the most recent testing for HIV (Table 2).

As for substance and alcohol use in the past 30 days, a higher proportion of female respondents reported marijuana use, although the difference with the male respondents was not significant. However, the difference was significant in alcohol use, with a higher proportion of female respondents consuming alcohol within the last 30 days compared to male respondents (*p* = 0.029). Not shown in the table are similar results in the past three months and lifetime exposure to alcohol and marijuana, but the differences were not significant. 

Twenty-eight percent (28%) of male and 34% of female respondents reported to have engaged in sexual activity while under the influence of either drugs or alcohol. A higher proportion of female respondents also reported engaging in sexual activity in the past 12 months not involving condoms. Conversely, male respondents reported a slightly higher rate of condom use during the most recent sexual encounter. However, the differences were also not significant. 

## 4. Discussion

### 4.1. Alcohol and Substance Use

Consistent with studies conducted in a college or university setting, the findings of the Get SMART survey support that the two most commonly abused substances in this demographic are alcohol and marijuana. 59% of the respondents reported having taken alcohol and 48% reported having smoked marijuana in the past three months. The rate of alcohol use is consistent with national studies, while the rate of marijuana use (39% to 43%) is higher compared to a national study reporting a 38% marijuana use among college students in the past 12 months. This result indicates the need for a substance use intervention, as marijuana, if used outside of a medical condition that warrants its use, is known to be a gateway drug for other illicit drugs such as cocaine and heroin. Studies conducted on the users of more potent illicit drugs reported marijuana use as the first drug used by an overwhelming majority of these users. With a high rate of alcohol drinking on campus, strategies to curb this behavior become paramount as alcohol not only impairs the sensorium but also clouds judgment, leading to engaging in high-risk behaviors such as drunk driving and random and unprotected sexual intercourse. 29% to 34% of respondents reported having sexual intercourse while under the influence of either alcohol or a substance. 

Comparing males and females, the latter have a significantly higher proportion of alcohol exposure. While contrary to most research indicating that more men consume alcohol than women, recent studies have shown that in young adults, more women tend to drink and consume higher volumes of alcohol when drinking in mixed gender settings, which is typical in college settings [25,26]. Interventions on alcoholic beverage drinking in colleges and universities may need to address the high proportion of female alcohol drinkers. Behavioral cues for drinking are different between males and females, necessitating tailored interventions rather than generalized approaches. Strategies to avoid the behavior will also be different, and an effective strategy for the males may not be effective for females. Evidence-based interventions for alcohol drinking (e.g., TIPS for the university) may need to be further enhanced and modified to accommodate gender-specific factors and behaviors to increase the likelihood of adopting health-protective and preventive behavior.

### 4.2. HIV Status and Testing Behavior

The high rate of non-use of condoms during the most recent sexual intercourse (41%) along with a low rate of knowledge of current HIV status (70%) creates a highly opportune milieu for HIV acquisition. The transmission chain of HIV cannot be eliminated if one is not aware of his or her HIV status. In the several occasions during the survey period where HIV testing opportunities were offered, only 24% of the respondents consented to the testing, while 76% (*n* = 187) declined HIV testing. The high proportion of respondents unaware of their HIV status (30%) and testing behavior are consistent with research on African American youth, this demographic having lower rates of knowledge on HIV status and even lower rates of testing compared to other racial and ethnic groups. Such behaviors increase the risk of transmitting HIV to others, particularly those in the African American community, who are more likely to prefer engaging in sexual activity within the same demographic. This may very well be the culprit for the higher rates of HIV among African American youth and young adults compared to any other racial group. 

While the levels of awareness of HIV status between male and female respondents were essentially no different from each other, the already low rates of HIV testing when offered and the even significantly lower testing rates among females reflect a behavior that needs to be addressed. Certainly, there are barriers, real or perceived, among the respondents that prevent HIV testing behavior or foster negative attitudes towards testing. Studies have shown that despite high doses of health education, awareness, and promotion, factors such as the environment, location, testing times, and individual perceptions all play a role in HIV testing. Privacy is a key component in HIV testing—and having testing booths and kiosks out in the public almost absolutely does not help, particularly among the female respondents of this survey. Locating testing sites in unconventional settings on campus, such as in the library or the chapel, may actually help mitigate the situation. While mobile vans provide an effective outreach tool to increase access to testing, the associated perception and stigma that mobile testing vans only test for HIV and STDs may deter individuals from actually utilizing the service. Hence, situating testing in unlikely locations and settings would probably increase the likelihood of being tested or being amenable to the opportunity. 

### 4.3. Condom Use 

There were two items in the survey that measured both preventive and risky sexual behavior, using a condom during the most recent sexual encounter and having unprotected sex within the last 12 months. Female respondents were more likely to report engaging in unprotected sex within the last 12 months and not using a condom during the most recent vaginal sexual activity. Although the differences between males and females were not significant, the low rates of condom use in both genders highlight the need for strategies that would increase utilization. There are several intrapersonal and interpersonal factors involved in this behavior, confounded by enabling environmental factors or the lack thereof. Intrapersonal perceptions that hinder condom use, which may be distinct between males and females, must be addressed in health promotion activities. Condom negotiation strategies are essential skills that can be taught to persuade couples to use condoms for protection. The environment can also be modified; in this case, the campus must nurture health in all aspects, including sexual health, by making resources, such as condoms, available and accessible, while promoting the behavior to increase acceptability. 

## 5. Conclusions

It is only logical to assume that lack of knowledge increases the risk of adverse health conditions due to engagement in risky behaviors. Therefore, it is a paradox that in institutions of higher education, the frequency of risky behaviors such as binge drinking, illicit drug use, and unprotected sex are among the highest. Health programs must take into account the unique environment of the college campus in the planning, implementation, and evaluation of interventions designed to curb risky behavior. Equally important to consider is the setting where the intervention is to occur. In the case of this HBCU’s urban location, where HIV prevalence is high, it is very likely that sexual behaviors are confined to the boundaries of the institution. Hence, prevention programs should not be limited to the campus perimeters and should include the community to have a meaningful and sustainable impact. 

In addition, tailoring interventions to address the unique needs inherent to factors such as gender will increase the likelihood of adapting and sustaining the preventive behavior. Particularly in a college setting where HIV is acquired primarily through sexual contact, it is paramount to identify and understand gender-specific beliefs, attitudes, perceptions, and practices that increase the predisposition to risky behavior. An in-depth qualitative study is recommended to further enrich the results of the survey and paint a more complete picture of the sexual health landscape of an educational institution. 

## Figures and Tables

**Table 1:** Demographic, substance use, and sexual behaviors of participants.

	Frequency	Percent Distribution
Gender (*n* = 365)		
Male	160	43.8%
Female	205	56.2%
Transgender	0	0%
Race/Ethnicity (*n* = 365)		
Black	347	95.6%
White	2	0.5%
Hispanic/Latino	6	1.7%
Others	4	1.1%
More than one race	4	1.1%
Sexual Orientation (*n* = 362)		
Heterosexual	304	84%
Gay/Lesbian	22	6.1%
Bisexual	27	7.4%
Unsure	9	2.5%
Relationship Status (*n* = 362)		
Single	277	76.5%
Married	7	1.9%
Divorced	2	0.6%
Committed Partner	64	17.7%
Casual Partner	12	3.3%
Lifetime Exposure (*n* = 365)		
Marijuana	225	62.3%
Prescription Drugs	30	8.3%
Cocaine	6	1.7%
Heroin	4	1.1%
Alcohol	262	72.6%
No Exposure	56	15.5%
Previous 3 Months Exposure (*n* = 365)		
Marijuana	172	48%
Prescription Drugs	12	3.4%
Cocaine	7	1.9%
Heroin	0	0%
Alcohol	210	58.7%
No Exposure	94	26.3%
Previous 30 Days Exposure (*n* = 365)		
Marijuana	148	41.3%
Prescription Drugs	12	3.4%
Cocaine	7	1.9%
Heroin	0	0%
Alcohol	192	53.6%
No Exposure	110	30.7%
Knowledge of HIV Status (*n* = 329)		
Yes	229	69.6%
No	100	30.4%
HIV Testing on Day of Survey (*n* = 187)		
Yes	44	23.5%
No	143	76.5%
Last Time Tested for HIV (*n* = 353)		
0–3 months	113	32%
4–6 months	45	12.8%
7–9 months	24	6.8%
>9 months	68	19.2%
Never Tested	103	29.2%
Ever Diagnosed with STD (*n* = 360)		
Yes	32	8.9%
No	328	91.1%
Used a Condom at Most Recent Sex (*n* = 365)		
Yes	214	58.6%
No	151	41.4%
Perceive Sex as Coping Response to Stress (*n* = 365)		
Yes	147	40.3%
No	218	59.7%

STD: sexually transmitted disease.

**Table 2:** Substance use and sexual behaviors by gender.

	Male	Female	*p* Value
Knowledge of HIV status			0.897
Yes	99 (69.2%)	130 (72.2%)	
No	44 (30.8%)	56 (27.8%)	
Tested for HIV on day of survey			<0.001
Yes	33 (36.3%)	11 (11.5%)	
No	58 (63.7%)	85 (88.5%)	
Last time tested for HIV			0.463
0–3 months	49 (31.0%)	64 (32.8%)	
4–6 months	16 (10.1%)	29 (14.9%)	
7–9 months	10 (6.3%)	14 (7.2%)	
>9 months	36 (22.8%)	32 (16.4%)	
Never tested	47 (29.8%)	56 (28.7%)	
Use of marijuana in the last 30 days			0.398
Yes	61 (38.8%)	87 (43.3%)	
No	96 (61.2%)	114 (56.7%)	
Alcohol drinking in the last 30 days			0.029
Yes	74 (47.1%)	98 (58.7%)	
No	83 (52.9%)	83 (41.3%)	
History of STD			0.697
Yes	13 (8.2%)	19 (9.4%)	
No	145 (91.8%)	183 (90.6%)	
Sex while under the influence of drugs/alcohol in the past 12 months			0.283
Yes	42 (28.4%)	63 (33.9%)	
No	106 (71.6%)	123 (66.1%)	
Sex without a condom in the past 12 months			0.406
Yes	72 (48.6%)	99 (53.2%)	
No	76 (51.4%)	87 (46.8%)	
Condom use in most recent sex encounter			0.906
Yes	49 (59.0%)	50 (58.1%)	
No	34 (41.0%)	36 (41.9%)	
